# Inflammatory Myofibroblastic Tumor of the Testis in a Patient With Cecal Carcinoma

**DOI:** 10.7759/cureus.44573

**Published:** 2023-09-02

**Authors:** R Chandramouli, Y Sree Sowmya, Abhishek Raghava KS, Debasis Gochhait, Pandjatcharam Jagadesan

**Affiliations:** 1 Radiation Oncology, Krishna Cancer Institute, Cuddalore, IND; 2 Radiation Oncology, Great Eastern Medical School and Hospital, Ragolu, IND; 3 Medical Oncology, Nizam's Institute of Medical Sciences, Hyderabad, IND; 4 Pathology, Jawaharlal Institute of Postgraduate Medical Education and Research, Puducherry, IND; 5 Radiation Oncology, Jawaharlal Institute of Postgraduate Medical Education and Research, Puducherry, IND

**Keywords:** carcinoma cecum, distant metastasis, local recurrence, benign tumor, myofibroblastic spindle cells, alk-1, vimentin, sma, fibroinflammatory disorders, inflammatory myofibroblastic tumor

## Abstract

Inflammatory myofibroblastic tumors (IMTs) are uncommon tumors that can be classified among fibroinflammatory disorders. IMTs are diagnosed after excluding all other entities, which can be considered differential diagnoses of IMTs. Microscopic examination of IMTs shows multiple myofibroblastic spindle cells, which are surrounded by inflammation. IMTs have lesser chances of progression to malignancy. The case defined below is one of the few cases in the literature that reports the presentation of IMT and another malignancy.

We describe a 72-year-old man who was found to have cecal carcinoma and later diagnosed with an IMT of the testis.

IMTs are generally benign tumors with a tendency for local recurrence. Patients affected by IMTs usually get diagnosed only after more than one biopsy. IMT is diagnosed only after ruling out other differential diagnoses. They rarely show invasiveness and metastasize. The presence of metastasis, recurrence, and other malignancies probably indicate poor prognosis and poor survival. The course of IMTs is usually benign, with good outcomes after surgery. IMTs have been known to recur, invade, or metastasize in sites such as paranasal sinuses, mediastinum, and the abdomen. In the case that we researched, vimentin and smooth muscle actin were strongly positive in the spindle-shaped cells, whereas anaplastic lymphoma kinase-1 was negative.

## Introduction

Inflammatory myofibroblastic tumors (IMTs) are uncommon tumors that can be classified among fibroinflammatory disorders. IMTs are diagnosed after excluding all other entities, which can be considered differential diagnoses of IMTs. Microscopic examination of IMTs shows multiple myofibroblastic spindle cells that are surrounded by inflammation. We describe a case of a 72-year-old man with a recurrent IMT of the testis and coexisting cecal carcinoma. The case reported here is one of the few cases in the literature that reports the presentation of an IMT along with another malignancy [1]. IMTs have lesser chances of progression to malignancy. The presence of another malignancy, namely, carcinoma caecum along with the IMTs, is a coincidental synchronous finding in this case. The etiology of IMTs can be unknown, genetic, trauma, infectious, or autoimmune. The occurrence of another cancer, cecum cancer, along with the IMT could have been due to genetic or autoimmune factors. 

This article was previously presented as an abstract at the conference of Indian Cancer Congress on November 8, 2017.

## Case presentation

**Figure 1:**
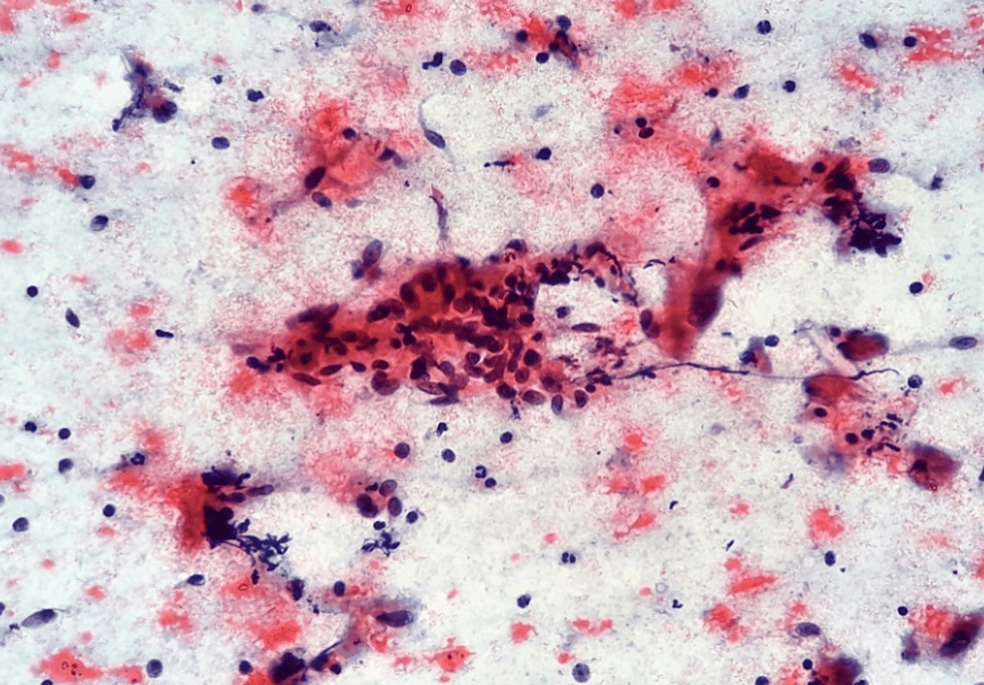
Papanicolaou stain (200X): Biopsy taken from paratesticular mass- IMT. Smear shows the spindle cells and some muscle cells along with numerous chronic inflammatory cells. IMT: Inflammatory myofibroblastic tumor

**Figure 2:**
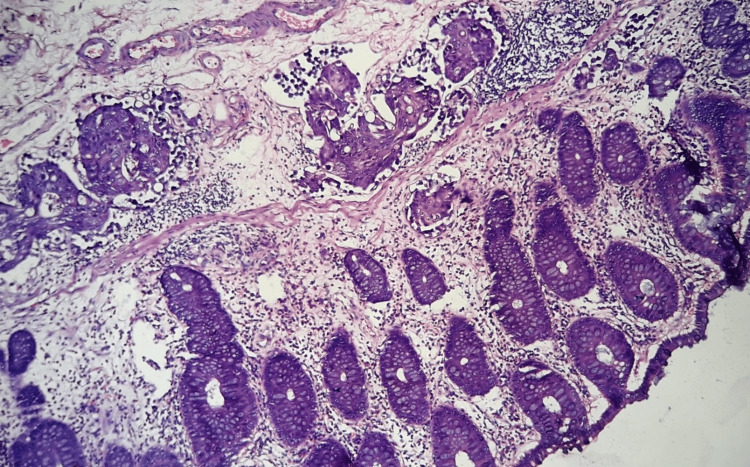
Hematoxylin and Eosin stain (200X): Biopsy taken from cecal mass. The section shows the colonic epithelium with the mucosa and submucosa showing many tumor cell clusters and lymphatic emboli.

A 72-year-old male, known smoker and alcoholic, presented with left scrotal swelling to an outside hospital. A 4*5 cm mass was present in the left scrotum. A testicular ultrasound was done and showed that the left scrotal mass was arising from the left epididymis and the right testicle was normal. The patient underwent a left-side high orchidectomy in October 2015. Postoperative histopathological examination showed a left paratesticular IMT, and the resection margins were negative (Figures 1, 2). 

He then presented to our hospital with recurrence after eight months in August 2016. His performance status was ECOG-2. On examination, there was a 22x10 cm mass in the left scrotal region, which was opined as unresectable. Slide review showed paratesticular IMT. Fine-needle aspiration cytology and biopsy from the perineal region showed a recurrent IMT, lymphoplasmocytic type with vimentin+, SMA + (smooth muscle actin), and ALK - (anaplastic lymphoma kinase).

**Figure 3:**
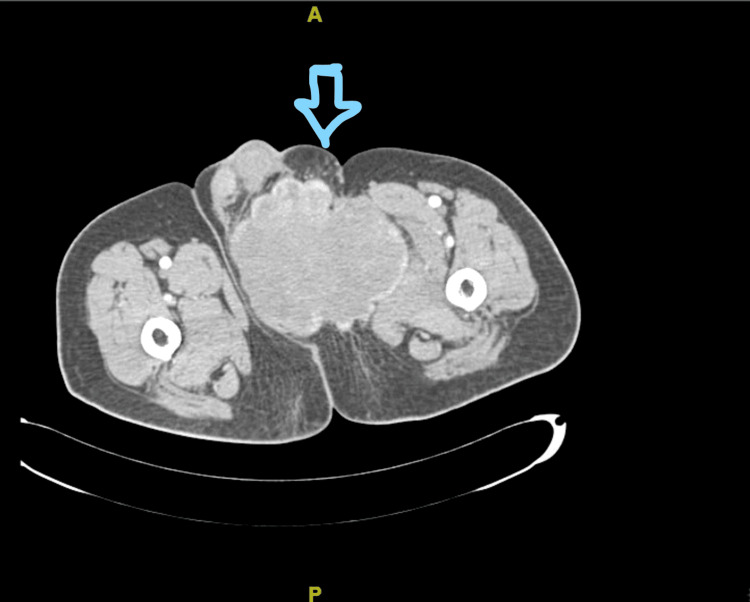
CECT section with a blue arrow pointing at the 11.6 x 9.4 x 11.4 cm sized partly defined lobulated heterogeneously enhanced soft tissue density lesion noted involving the inguinoscrotal region on the left side CECT: Contrast-enhanced computerized tomography

**Figure 4:**
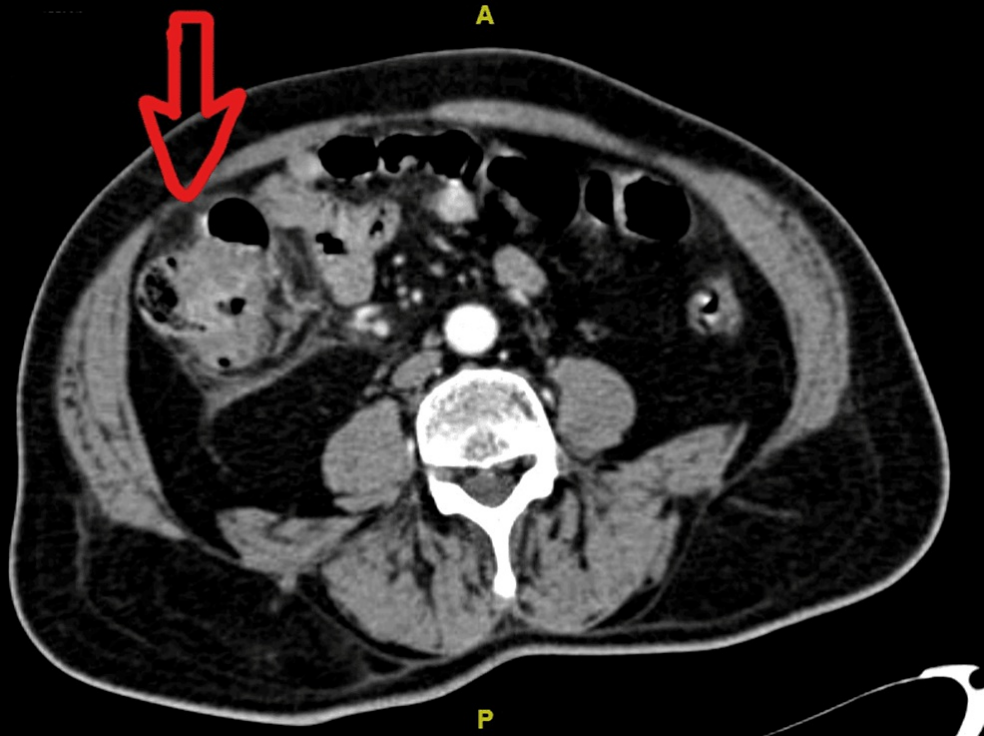
CECT section showing 2.5 x 2.3 cm sized focal enhancing thickening involving the anterior wall of the cecum CECT scan: Contrast-enhanced computerized tomography scan

On evaluation for recurrence, contrast-enhanced computerized tomography (CECT) thorax, abdomen, and pelvis report showed 2.5 x 2.3 cm sized focal enhancing thickening involving the anterior wall of the caecum. A 11.6 x 9.4 x 11.4 cm sized partly defined lobulated heterogeneously enhancing soft tissue density lesion was noted involving the inguinoscrotal region on the left side with scrotal skin involvement. Inferiorly, the lesion was seen extending up to the level of the left side root of the scrotum. Medially, the lesion was seen infiltrating into the root of the penis and infiltrating it to the right side. The lesion was seen infiltrating into the adductor muscles, pectineus, obturator externus, and gracilis muscles on the left side. The lesion was seen closely abutting the pubic symphysis, left superior and inferior pubic rami with loss of fat plane. There were two other enhancing soft tissue density lesions noted in the root of the right scrotum, the largest measuring 2.5 x 2.5 cm (Figures 3, 4). 

Colonoscopy-guided biopsy revealed adenocarcinoma, well differentiated. Carcinoembryonic antigen was elevated. The patient underwent a right hemicolectomy. Postoperative biopsy showed adenocarcinoma- moderately differentiated, 15 pericolic lymph nodes with a tumor, with extranodal extension present, pStage IIIC pT3N2bM0. Palliative radiotherapy 20 Gy/ 5 Fr was delivered to the left paratesticular mass, and the patient had stable disease on response assessment. The patient had significant symptomatic improvement with respect to pain. The pain decreased in intensity from moderate intensity of pain to mild intensity of pain after radiotherapy. The patient was not fit for chemotherapy due to poor performance status- ECOG-3 and was put on the best supportive care. The patient developed breathlessness two months after the last fraction of radiotherapy. Imaging showed lung metastasis. Lung metastasis could have arisen from either an IMT or carcinoma cecum. The patient was then put under the best supportive care and passed away in April 2017, one and a half years after he was diagnosed with malignancy. 

## Discussion

IMTs are composed of plasma cells, lymphocytes, myofibroblasts, fibroblasts, and histiocytes. Injury results in the release of inflammatory mediators such as cytokines and interleukin-1. Inflammatory mediators cause fibroblast proliferation, endothelium becoming leaky and prothrombotic, and neutrophil extravasation.

The exact cause of IMTs is not clear. Probable risk factors can be infection, adenoidectomy, trivial injuries, and smoking.

Diagnosis of IMTs can be reached after distinguishing them from other similar conditions like benign tumors, reactive tumors, spindle cell tumors including carcinoma, leiomyoma, nodular fasciitis, peripheral nerve sheath tumor, and low-grade myofibroblastic sarcoma [2].

The treatment of choice is surgical resection. Other modalities include radiotherapy, crizotinib, steroids, and methotrexate. Tumor recurrence after surgery was found to be as much as 25-37% in extra-pulmonary IMTs and up to 85% in abdominopelvic IMTs [1].

The most common cause of paratesticular mass is adenomatoid tumors followed by IMTs. Roughly 6 % of all paratesticular masses are paratesticular IMTs [3]. IMTs are usually indolent, with a prolonged five-year survival of 91.3% [4]. The rate of malignant transformation is less than 5 % [5]. The rate of distant metastasis is approximately 11% [6].

The most common sites of metastasis are the lungs and brain, followed by the liver and bones [7]. IMTs are radiosensitive and complete response with no local recurrence during follow-up in around 2/3rd of the cases that were studied has been shown in various studies [8,9].

Adjuvant radiotherapy in cases with microscopic or macroscopic residual tumors after surgical resection has been shown to reduce the rate of local recurrence [10].

## Conclusions

Though IMTs are benign, they can locally erode the nearby structures and hence have to be operated on as early as possible to provide cure and symptom relief. Early diagnosis results in a high chance of complete resection, prevents local complications, and reduces the chance of malignant transformation and distant metastasis. Surgical resection is the treatment of choice and gives an accurate histopathological diagnosis in many cases. 

Histopathological examination with IHC is crucial for establishing the diagnosis. IHC helps exclude the other benign and malignant tumors that may mimic IMTs. 
